# Some Clinical Cases

**Published:** 1893-03

**Authors:** A. G. Allan

**Affiliations:** Philadelphia, Pa.


### SOME CLINICAL CASES.

A. G. Allan, M. D., Philadelphia, Pa.

In presenting to you the following clinical cases I have had but two objects in view: 1st. To show the efficacy of our system in dealing with eye symptoms. 2d. That the highest potencies act immediately and, I may say, almost like magic in acute cases. These cases are such as every one of us is liable to meet with in daily practice; so I consider them to be of more practical value in a paper like this than a report of cases such as seldom occur, or where the writer has had the good fortune to rescue an unfortunate from the verge of the grave. While cases of such a nature may appear wonderful and call forth encomiums from a circle of admirers, unfortunately they usually contain very little of value to the student of Homœopathy.

Case I. September 10th, 1891.—Miss A., aged five years, was brought to me by her mother to have her eye treated. She had a central phlycten on the cornea of the left eye; the globe of the eye was inflamed, but the redness was not as marked as we should have expected from a phlycten in that situation. There were present some photophobia and lachrymation, but these were not very marked, as the child was able to open her eyes, although facing a window, and when I separated the lids to obtain a better view of the eyeball there was no spasm of them, which is often so marked in corneal troubles. Of course the child was too young to give her subjective symptoms, so we were forced to rely solely upon the objective ones. Her mother said that this followed an attack of scarlet fever two years ago. Before this attack of fever the child had never had sore eyes, but that since then she had had eye troubles almost continuously. It began in the left eye and had always been worse in that one, though at times the other one was attacked too. The only other symptom that her mother observed was that the eyelids adhered by night so that she had to bathe them in the morning before they could be opened. In studying this case for a remedy we must not lose sight of the fact that the eye and ear symptoms were concomitant, as this will assist us very much in making our choice. If we turn to page 207 of Berridge's *Repertory* we shall find under the rubric *Cornea, Eruptions, Ulcers,* Calc-c. And under *Color Red,* also Calc-c. We also shall find that Calc-c. has adhesion of the lids by night and that Calc-c. in eye troubles prefers the left side. Accordingly she received a single dose of Calc-c.$^{13m}$ with instructions to report in two days.

September 12th, 1891.—Eyeball not so red; lids did not adhere this morning. Her mother does not think that there is as much discharge from the ear as there was before she came for treatment.

September 22d, 1891.—Did not see patient again until this date. Her mother stated that as she continued to improve that she did not think it was worth while to come back. The inflammation of the eye had all disappeared by the next day and nothing was visible now to indicate that there had been any eye trouble except a slight haze in the cornea caused by repeated attacks of keratitis. The ear had not discharged since the last visit, and an examination revealed the presence of but a slight moisture at the bottom of the external auditory canal. The mother was told to bring the child back at the end of a week, but as she never returned it is reasonable to suppose that she recovered and that her eyes have remained well.

CASE II. March 27th, 1892.—Miss C., aged thirteen, came to have her left eye treated for an attack of phlyctenular conjunctivitis. There were several well-developed phlyctens on the conjunctiva close to the border of the cornea; but none on the cornea. The child complained of itching and burning, which was ameliorated by rubbing the eye. The sight was impaired; it seemed as if she were looking through a fog. This was ameliorated by rubbing the eye, and aggravated by bodily exercise and reading. The eye was sensitive to the light. The child had not yet menstruated; she was timid, and her mother said "her feelings are very easily hurt, and she cries at almost nothing." Her mother also said that she often complained of the room feeling close when it did not seem so to others. These general symptoms pointed strongly to Pulsatilla, and, as the eye symptoms are also those of Puls., as may be seen by referring to the *Materia Medica*, she received a single dose of Puls.$^{55m}$. She improved rapidly under this dose, so that at the end of a week, when she returned again, her eye appeared perfectly well.

On April 3d, 1892, I was sent for, as she was too sick to go out of the house. I found her suffering from an attack of diphtheria. Her skin was hot and dry, face flushed, pulse 127, throat swollen and sensitive to touch, great difficulty in swallowing, with the false membrane developed on both tonsils and the soft palate. The left side hurt her more than the right, and the left tonsil appeared to be more swollen and have more membrane on it than did the right. For this she received a single dose of Lach.$^{mm}$. It caused an amelioration in the first twelve hours, the pulse falling to 98, and the skin not feeling nearly as hot as it did in the morning. The next morning, the 4th instant, I found her pulse 102, with no apparent change in any of her other symptoms, either subjective or objective, so I decided to repeat Lach. I considered it best to change the potency, so I gave her a dose of the CM potency. This was followed by a marked improvement, which continued until the 6th instant. The pain during deglutition and the sensitiveness of the throat disappeared almost entirely, the pulse fell to 90, and the false membrane disappeared from all parts of the throat except the left tonsil, which still remained swollen, with some small patches on it. Again I decided that it would be best to repeat, so I gave her a single dose of Lach.$^{41m}$, and by evening all her symptoms were gone. There was now no trace of membrane on the throat anywhere; she could swallow the same as she did before the attack, the skin felt cool and natural, and her pulse was 78, even slower than I would have looked for it to be in a child of her age. With this ended her attack of diphtheria, but not all her troubles, for on the 12th instant she had another attack of phlyctenular conjunctivitis in the left eye, in appearance and symptoms being the same as the attack of March 27th. For this she received a single dose of Puls.$^{cm}$. It promptly relieved, the eye again looking well within a week. Up to the present time there has been no return of the eye symptoms, and since then she has not had any other sick spell or taken any other medicine.

CASE III. December 30th, 1891.—Miss D., aged seventeen, came for treatment with a central ulcer of the left cornea. The ulcer was large and very deep and the cornea seemed to bulge at that point, making it appear as if a perforation was imminent. This attack was of nearly three weeks' duration, but she had had a previous attack about five years ago from which she had recovered very slowly under old-school treatment. The following were her symptoms: Eyeball very red, the cornea vascular and very much infiltrated. She complained of considerable pain, which was usually worse in the morning on rising, apparently caused by the light, worse in the afternoon and at night, worse from the heat of fire. Burns and itches; sensation like sand in the eye when winking; sensitive and painful to touch; pains shooting. Profuse lachrymation, tears feel as if they would scald the eye; great photophobia. Lids swollen, red, and feel heavy. For this she received a powder of Bell.$^{cm}$, to be dissolved in water, taken in six doses half an hour apart. Took first dose at eleven o'clock A. M.

December 31st, 1891.—Eye felt worse, with more shooting pains. The burning, itching sensation like sand under the upper lid when winking and the lachrymation were also worse after taking the medicine until ten P. M., when she fell asleep. She slept some in short naps the rest of the night but the next day after being up and about awhile the eye felt much easier.

January 2d, 1892.—Yesterday and to-day the eye felt much better. She did not have the same aggravation when first getting up. The eyeball is not so red, ulcer not so deep. Cornea does not appear to bulge at the point of ulceration. Vessels on cornea scarcely visible.

January 5th, 1892.—Eye much better in every respect. Ulcer much smaller. Vessels on cornea only visible when using a lens and there are only a few about the ulcer. Only a very little redness of the eyeball; but on the conjunctiva below the ulcer close to the margin of the cornea a large phlycten has made its appearance.

January 8th, 1892.—Cornea improved, can see no vessels on it; ulcer nearly all healed. Another small phlycten has developed on the conjunctiva at the inner margin of the cornea. Bell.$^{cm}$, single dose.

January 11th, 1892.—Eye same as when last here. No change in any respect. Merc.-sol.$^{cm}$, one dose.

January 14th, 1892.—Improved; less redness of the eyeball. Cornea clearer, vision improving, can see $\frac{20}{100}$.

January 18th, 1892.—The phlyctenules have disappeared. No redness of conjunctiva. Vision same as last visit. Cornea clouded at the point of ulceration.

January 30th, 1892.—Eye has remained well since she was here last. No more phlyctens have appeared. Cornea much clearer, but where the ulcer was there is a small macular left.

This girl continued to improve without any more medicine, and the only trouble left in the eye is a very small scar to mark the spot where the ulcer was. Her vision is now $\frac{20}{40}$.

---

